# Scaling up genetic circuit design for cellular computing: advances and prospects

**DOI:** 10.1007/s11047-018-9715-9

**Published:** 2018-10-05

**Authors:** Yiyu Xiang, Neil Dalchau, Baojun Wang

**Affiliations:** 1 School of Biological Sciences, University of Edinburgh, Edinburgh, EH9 3FF UK; 2 Centre for Synthetic and Systems Biology, University of Edinburgh, Edinburgh, EH9 3JR UK; 3 Microsoft Research, Cambridge, CB1 2FB UK

**Keywords:** Cellular computing, Synthetic biology, Genetic circuit, Genetic logic gates, Analog computation, Biodesign automation

## Abstract

Synthetic biology aims to engineer and redesign biological systems for useful real-world applications in biomanufacturing, biosensing and biotherapy following a typical design-build-test cycle. Inspired from computer science and electronics, synthetic gene circuits have been designed to exhibit control over the flow of information in biological systems. Two types are Boolean logic inspired TRUE or FALSE digital logic and graded analog computation. Key principles for gene circuit engineering include modularity, orthogonality, predictability and reliability. Initial circuits in the field were small and hampered by a lack of modular and orthogonal components, however in recent years the library of available parts has increased vastly. New tools for high throughput DNA assembly and characterization have been developed enabling rapid prototyping, systematic in situ characterization, as well as automated design and assembly of circuits. Recently implemented computing paradigms in circuit memory and distributed computing using cell consortia will also be discussed. Finally, we will examine existing challenges in building predictable large-scale circuits including modularity, context dependency and metabolic burden as well as tools and methods used to resolve them. These new trends and techniques have the potential to accelerate design of larger gene circuits and result in an increase in our basic understanding of circuit and host behaviour.

## Introduction

In order to survive and reproduce, cells must sense a wide variety of inputs both external and internal. In response they compute and actuate a number of output functions such as changes to cell morphology (Goranov et al. 2013), or production of proteins and small molecules (Williams et al. 2016). We can exploit these changes for desirable functions such as the manufacture of valuable products or sensing of dangerous environmental toxins (Ro et al. 2006; Wang et al. 2013a; Bereza-Malcolm et al. 2015; Machado et al. 2016; Bernard and Wang 2017). Many of these properties have useful real-world functions and it is desirable to manipulate them for our own goals.

Historically, the approach to the workflow in biotechnology has been based on bespoke, unique solutions. Often this results in laborious ad-hoc laboratory processes which result in an inability to port solutions from one problem to another, and a missed opportunity to retain valuable information learned through the process. There exists no one precise universally accepted definition of synthetic biology although most do overlap strongly. One definition states that synthetic biology is ‘the design and engineering of biologically based parts, novel devices and systems as a well as the redesign of existing, natural biological systems,’ (Clarke and Kitney 2016). Broadly speaking, synthetic biology is a rational approach to biotechnology inspired by ideas from engineering and aims to make designing biology easier, faster and more predictable. Key concepts in this pursuit are standardization, modularity, characterization and orthogonality (Andrianantoandro et al. 2006). The field has attempted to create libraries and repositories of biological parts (Knight 2003), and incorporated the engineering design-build-test-learn (DBTL) workflow in various parts of the literature (Paddon and Keasling 2014; Hutchison et al. 2016; Clarke and Kitney 2016; Cox et al. 2018), industry (Anne Ravanona 2015; Siliconreview Team 2017), and government (Si and Zhao 2016). Ideally one could dependably generate entirely new systems with novel functions and predictable behaviour from a standardized parts list.

A major focus in the field has been on cellular computing, in which response pathways can be co-opted to produce useful biological computing devices which can produce programmable and predictable outputs in response to diverse input signals. One design paradigm has been to use gene circuits to regulate behaviour, of which two methods are digital binary-like and analog models. Other models such as DNA computing through tiling, hybridisation, self-assembly or recombination exist, but this review will focus on genetic circuits. A review covering other aspects of computing with biological parts was done by Moe-behrens (2013).

**Fig. 1:**
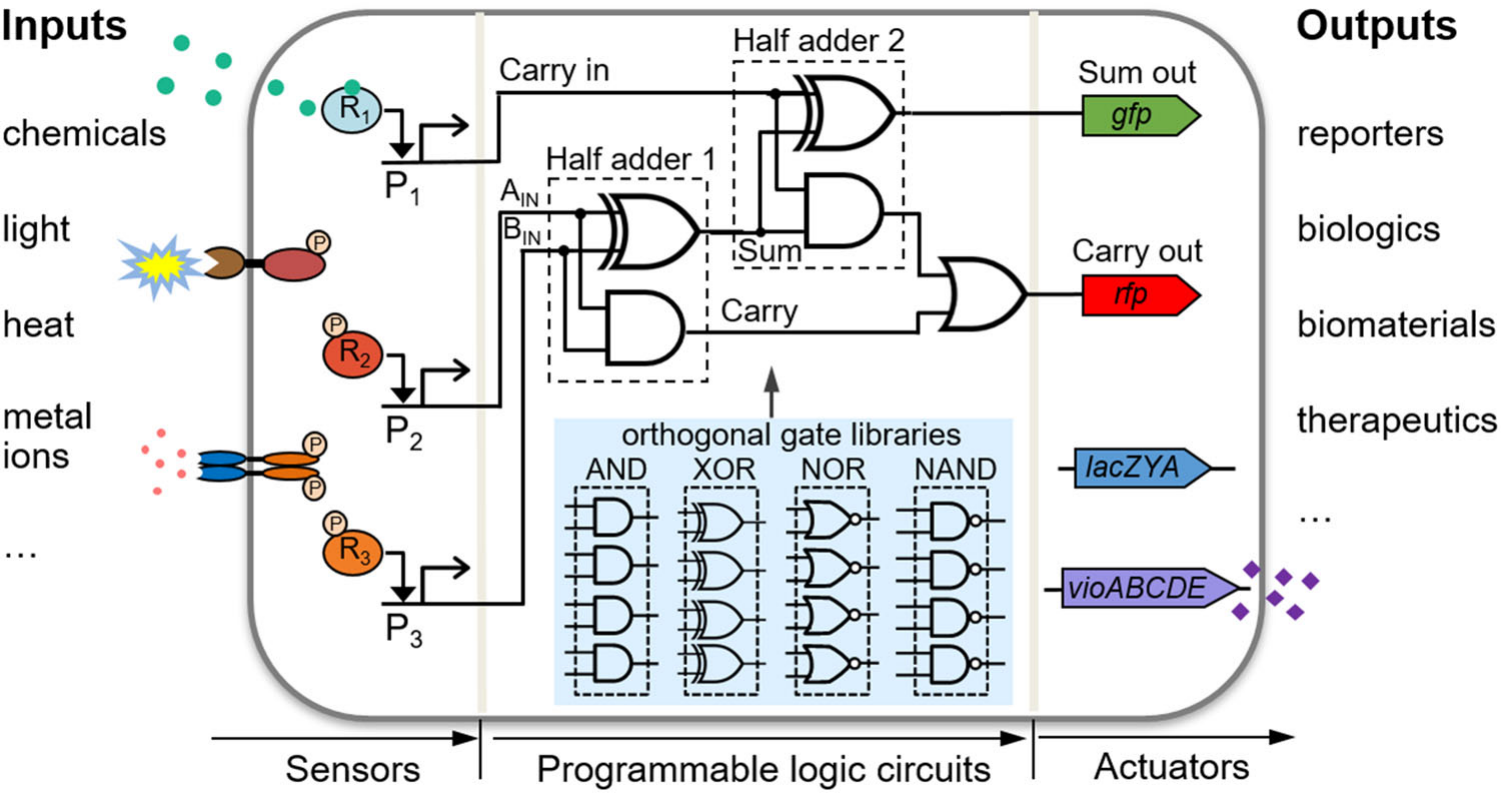
Programmable cellular computation with scalable signal processing capacity. To achieve large-scale control of cellular behaviour, an expanded library of versatile orthogonal genetic regulatory blocks and associated wiring principles are needed. For example, a genetic 1-bit full adder program adds binary numbers, it has 3 inputs and 2 outputs, and can be constructed from 5 modular logic gates that are wired in 3 layers and selected from well-characterized orthogonal gate libraries. The genetic circuits can be coupled to modular input genetic sensors and output actuators to achieve complex decision making for a variety of human desired applications

**Fig. 2:**
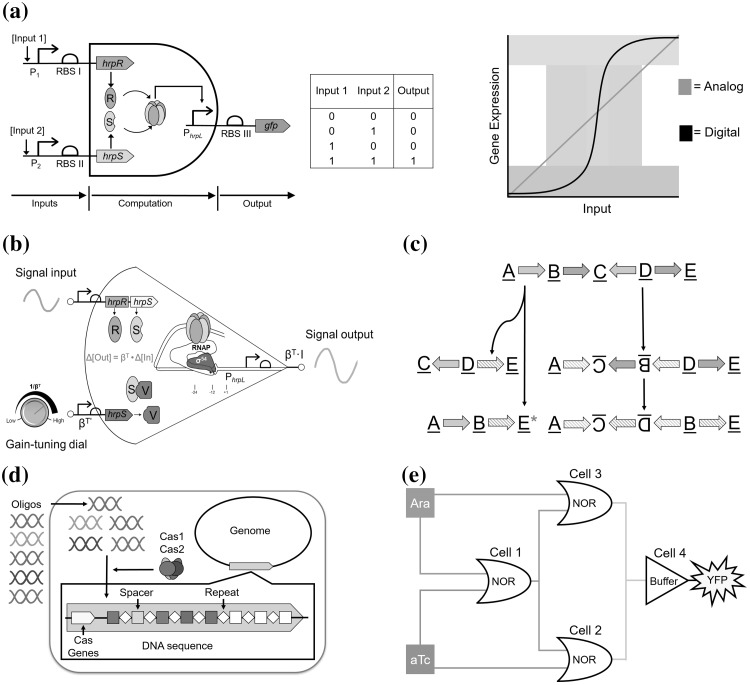
Versatile cellular computing paradigms enabled by synthetic biology. **a** A two-input AND gate using the σ^54^-dependent HrpR/HrpS hetero-regulation module and the corresponding truth table. The HrpS and HrpR enhancer-binding proteins expressed from separate inducible input promoters bind to form a heteromeric complex which activates the output σ^54^-dependent *hrpL* promoter. Also shown is a graph with representative transfer functions of digital (grey) and analog (green) signal responses. Digital signals have a steep sigmoidal response with a large change in expression over a small change in input. Analog signals have a much more graded response. **b** An analog transcriptional signal amplifier designed based on the *hrp* gene regulation module from plant pathogen *P. syringae*. The amplifier achieves different gains and input dynamic ranges by varying the expression levels of the underlying ligand-free ultrasensitive activator proteins (HrpRS) in the device. HrpV binds and sequesters HrpS so that it can no longer bind its co-activator HrpR and is used to modulate the intensity of the amplified signal output. **c** A recombinase-based state machine. Depending on if the att sites face in the same direction or towards each other, recombinases can excise or invert pieces of DNA respectively. By doing so they can record events, and with the correct elements can modulate gene expression. Striped arrows represent post inversion, the new site is sequentially different from the old one. **d** CRISPR-based memory storage. Oligos are sequentially incorporated into CRISPR arrays in the genome by the cas1–cas2 complex. Each oligo encodes information in the DNA sequence. The sequence at which oligos are ordered can be controlled by spacing addition over time. **e** Spatially distributed gates can reuse parts and signalling molecules, using proximity as a way to insulate signal propagation. Here 3 NOR gates effectively replicate a XOR gate using the inducers arabinose (Ara) and anhydrotetracycline (aTc). Signal lines green and yellow represent the quorum sensing molecules *N*-3-oxo-dodecanoyl-homoserine lactone (3OC12-HSL) and *N*-butyryl-homoserine lactone (C4-HSL) respectively and the output is yellow fluorescent protein (YFP). (Color figure online)

Digital-like biological parts often resemble logic gates and their discrete binary states found in silicon transistors such as the 1-bit full adder; comprising 5 logic gates wired in 3 layers with 3 inputs and 2 outputs (Fig. 1). Here there is a high contrast between high levels and low levels of output, corresponding to a discrete binary ON or OFF state respectively. As seen in Fig. 2a this means a high and sharp change in the output signal over a small change in the input signal once it hits a threshold level (represented by a hill coefficient > 1). Often these circuits will be described by Boolean logic, in which all values are reduced to either TRUE (1) or FALSE (0) (Bradley et al. 2016a). Logic operations like AND (where output is TRUE only if both inputs are TRUE), can be represented by a Boolean truth table as well as circuit symbols adapted from electronics as shown in Fig. 2a. Ideally, both the input and the output must be able to be connected to and interact with upstream and downstream components and operate in the intended fashion (be modular), the signal output must be stable, exhibit low noise (random unintended fluctuations), and have a large ON:OFF ratio, or dynamic range (Bradley and Wang 2015). This prevents the signal from being degraded as it propagates through a system. Digital logic is particularly useful in a decision-making circuit such as in natural cell differentiation or apoptosis. The strong state change is ideal for reliable state transitions and signal integration as digital circuits are relatively robust to noise. Early instances included the gene toggle switch (Gardner et al. 2000), the repressilator (Elowitz and Leibler 2000), and the autoregulator (Becskei and Serrano 2000). From there on, binary-like logic gates such as AND (Anderson et al. 2007; Wang et al. 2011), OR (Mayo et al. 2006), and NOR (Guet 2002; Tamsir et al. 2011) were built and combined into more complex circuits, for instance a 4 input AND gate (Moon et al. 2012).

In contrast, analog responses are designed to give a continuous output changing dynamically according to the input. Good transfer functions of analog computation are clear and well defined, responding to a large range of input, as well as exhibiting low noise and being reliable and modular, again as shown in Fig. 2a. Analog computation is particularly resource efficient, good at generating small autonomous responses to temporal differences, autoregulation and interaction with host metabolism, although analog circuits are susceptible to disruption from noise and temporary perturbations in input (Sarpeshkar 2014). Examples of analog circuits include amplifiers for scaling transcriptional signals in cascaded gene networks (Wang et al. 2014), comparators that convert signals from analog to digital (Rubens et al. 2016) and networks that allow for reconfigurable inversion of the network transfer function (Lewis et al. 2018).

Potential applications for gene circuits have been hinted at with the proof-of-concept arsenic biosensors (Wang et al. 2013a), and the cancer killing 3-input NOR gate “classifier circuit” (Xie et al. 2011). Cell consortia have been used to build an analog to digital converter detecting smell using olfactory receptors (Müller et al. 2017) whilst memory engineered commensal *E. coli* was able to function for six months in a mouse gut, sensing and reporting on the presence of inflammation-indicating tetrathionate (Riglar et al. 2017).

Many of the early examples were small in scale and design, often with few synthetic parts. Gander et al. (2017) reviewed the literature and found some of the largest circuits in recent publications only had 7 parts and 6 connections. This is roughly in line with Privman et al. (2008) who surmised that under optimal conditions going over 10 processing steps would be difficult using methods available at the time and would require new noise reduction paradigms. Recently larger circuit examples have been emerging, advances in characterization techniques and the expansion of the library of functional parts has promised a much larger space in which to build more complex circuits. RNA based parts enabled construction of a substantial 12-input disjunctive normal form circuit (Green et al. 2017), whilst a 6-input 1-output Boolean logic look-up table circuit was also demonstrated in mammalian cells. The group created a circuit which receives 4 selection inputs and 2 data inputs, the selection inputs determine which one of sixteen 2 data input logic functions the circuit uses allowing switching of logic in cells on the fly. By normalizing the promoter and recombinases used, they were able to generate 113 circuits, of which 109 were working, the largest collection of functionally unique logic circuits in mammalian cells as of publication (Weinberg et al. 2017). Another large circuit, the 1-bit full adder was functionally constructed in mammalian consortia incorporating 22 separate gates distributed amongst 9 specialized cell types in a complex three-dimensional environment (Ausländer et al. 2017). However construction of such large scale genetic circuits are uncommon, large numbers of logic gates in single cells are scarce and require significant amounts of time and effort to work through an iteration of the design-build-test-learn cycle.

In this article, we will discuss the tools and challenges surrounding the construction of large-scale gene circuits. We will decompose this into the DBTL cycle for clarity: design—the arrangement of reusable components to produce biological programs, build—large scale DNA assembly, test—high throughput characterization and debugging tools, learn—modelling and circuit design automation. We will review common methods enabling control of genetic circuitry and DNA assembly. The latest in advanced characterization and debugging methods using cell free systems, microfluidics, and ribonucleic acid (RNA) sequencing (RNA-seq) will be discussed. We will give insights into automated gene circuit design software and examine the implementation of more complex computing paradigms such as distributed computing and memory integration. Although most of the work has been directed towards genetic circuits in single cells, there has been a significant body of work that has experimented with using cell consortia, separating out circuits into many hosts (Regot et al. 2011; Macía et al. 2012). Integration of memory enables a move away from just combinatorial logic (in which the output is a function of the present inputs), making sequential logic possible (Siuti et al. 2013; Purcell and Lu 2014; Roquet et al. 2016). The challenges in scaling up circuit design and the techniques used to tackle them will be discussed. Focus will be on the obstacles to modularity as well as context effects and metabolic burden.

## Design: expanded toolbox for engineering complex gene regulation programs

**Fig. 3:**
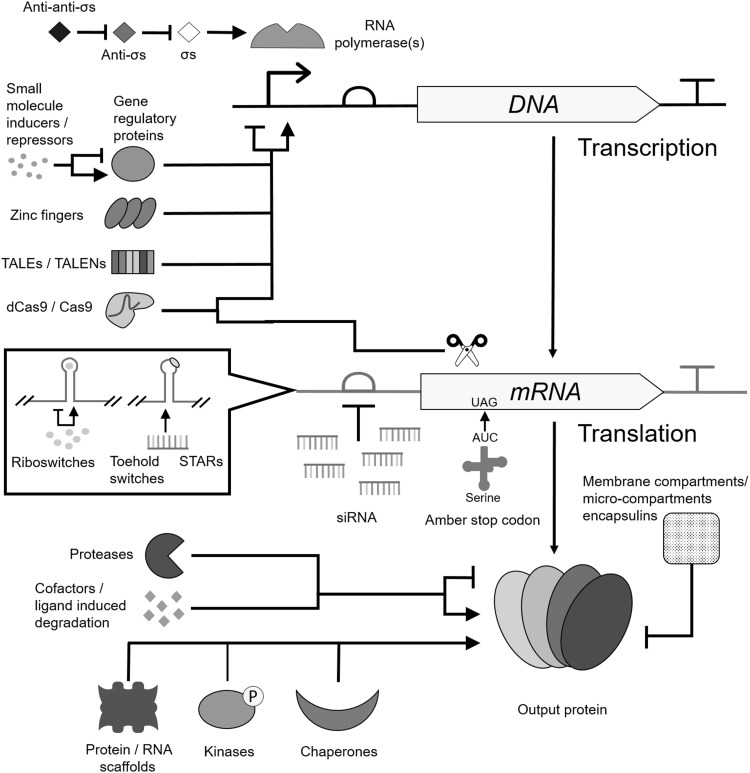
Expanded toolbox for engineering complex gene regulation programs. These include using proteins that affect DNA transcription and RNA translation through protein-DNA and protein-RNA base pair binding. Also shown is the ability to use RNA secondary structure and base pair binding to control mRNA translation initiation. Protein activity can also be controlled by other proteins, through protein–protein interactions or enzymatic reactions that modulate activity. The activity of many regulators can be controlled by small molecule ligands/cofactors. (σ = sigma factors, STARs = small transcriptional activating RNAs, siRNA = small interfering RNA, TALE(N)s = transcription activator-like effector (nuclease)

The construction of any large-scale circuit requires a large library of well characterized, orthogonal and modular gates comprising the ‘building blocks’ of the system. Since the beginning of the field there has been a significant and promising expansion of the molecular toolbox. A large variety of repurposed biochemical tools have been demonstrated to admit some degree of control over cellular state. Many of these tools co-opt biology’s central dogma, the expression of a gene and the information flow from DNA to RNA to proteins. These can be broadly grouped into three types; control of DNA transcription, messenger RNA (mRNA) translation or protein–protein interactions. Many of these methods are shown in Fig. 3.

Inducible parts provide useful externally mediated control of systems whilst customizable DNA sequence binding enables a significant expansion of the number of orthogonal parts available for large circuits (Garg et al. 2012; Lohmueller et al. 2012; Qi et al. 2013; Kiani et al. 2014; Nielsen and Voigt 2014; Li et al. 2015).

Control of expression using RNA tends to be less burdensome on host metabolism and can be governed by cleavage (Qi et al. 2012), pair binding (Rinaudo et al. 2007; Anderson et al. 2007; Xie et al. 2011; Wroblewska et al. 2015) or secondary structure (Sharma et al. 2008; Auslander and Fussenegger 2014; Myhrvold and Silver 2015; Chappell et al. 2015; Karagiannis et al. 2016), as folding has been proved to be moderately predictable using software such as NUPACK (Zadeh et al. 2011; Wang and Buck 2012). Protein–protein interactions such as those in the *hrp* (hypersensitive response and pathogenicity) gene regulation system can be utilized to generate versatile multi-input genetic logic gates (Wang et al. 2011; Wang and Buck 2014). Artificially or naturally split inteins can integrate signals (Schaerli et al. 2014) and different proteins can even be fused to each other in order to have hybrid properties (Wang et al. 2013b).

The expansion of parts has been enabled by a variety of tools; genomic part mining was successful in building a library of 16 orthogonal strongly repressing TetR family repressors (Stanton et al. 2013), 4 different T7 polymerases (Temme et al. 2012) and 20 different sigma factors (Rhodius et al. 2013). Chen et al. (2013) characterized 582 terminators whilst a protein engineering approach using bioinformatics and site directed mutagenesis generated different DNA binding specificities in the cAMP receptor protein (CRP) family (Desai et al. 2009). However, it is likely that the predictable natures of customizable DNA binding proteins and RNA secondary structure-based tools will provide the largest number of orthogonal parts. Didovyk et al. (2016) generated 633 possible orthogonal (to host and circuit) guide RNA’s for CRISPR/Cas9 mediated gene regulation through computational screening whilst 180 TALE effectors were designed by Garg et al. (2012). Other studies developed 26 toehold switches (Green et al. 2014), 4 RNA riboregulator/genetic switchboard pairs (Callura et al. 2012), 6 RNA-IN-RNA-OUT families (Mutalik et al. 2012), 5 zinc fingers (Khalil et al. 2012), and 20 sigma factors (Rhodius et al. 2013). From here, one can determine that there is a significant number of parts, far more than is needed than for most current circuits and yet circuit size has not increased in proportion to the size of the library.

Finally, there is a variety of tuning ‘knobs’ which although not able to provide control themselves, can be used to adjust the dynamic properties of a system to respond effectively to expected inputs and produce desired outputs depending on the need. These include changing the strength of the promoter sequence, hybrid combinations of promoter sequences (Chen et al. 2018), operator site modification (Ang et al. 2013), ribosome binding site (RBS) modification (Salis et al. 2009), altering plasmid copy number (Guido et al. 2006), using decoy DNA operators (Lee and Maheshri 2012), RNA interference (RNAi), degradation tags (Bonger et al. 2011), or co-expression with sequestering proteins or molecules (Wang et al. 2014). Cooperativity has been improved using oligomerization domains (Hou et al. 2018). Positive feedback loops and signal cascades have improved the ON/OFF ratio in digital-like circuits which is often poor due to an inherent basal level of ‘leaky’ gene expression even without the presence of activators or in the presence of repressors (Bradley et al. 2016a). A informative review of available control and tuning methods was covered by Ang et al. (2013), Bradley and Wang (2015) and Bradley et al. (2016b).

## Build: standard large-scale DNA assembly

Although genes can be chemically synthesized, it is still an expensive solution despite the recent drop in cost and importantly does not incorporate any standardization. Ideally all parts would be characterized, stored in a library and then manipulated at will using a common scalable DNA assembly protocol. Initial attempts were based on lengthy stepwise restriction enzyme mechanisms such as in BioBricks™ (Shetty et al. 2008). The latest methods are termed ‘one pot’ as multiple fragments can be assembled at once in a defined order saving many man-hours in the laboratory. These include Golden Gate and its derivations; MoClo (modular cloning) and Goldenbraid (Engler et al. 2008; Sarrion-Perdigones et al. 2011; Weber et al. 2011). Gibson assembly (2009), the most popular non-synthesis based method (Kahl and Endy 2013), uses homology of overlapping single stranded DNA which also avoids the necessity of removing forbidden sequences (such as restriction enzyme sites) in the sequences being assembled. Unfortunately, the superseding of BioBricks with formats such as Gibson assembly has moved parts back towards non-modular tailored solutions. Assembled parts cannot be ported to another assembly without new specific primer, and each new PCR reaction itself has bespoke conditions affecting assembly success due to the lack of standardization in flanking sequences. This can also have ramifications in terms of genetic context (discussed later on), and libraries of parts become more complex to reuse. Efforts have been made to add modular prefixes and suffixes; Casini et al. (2014) developed a strategy named MODAL (Modular Overlap-Directed Assembly with Linkers) and similar methods were published elsewhere (Torella et al. 2014). Woodruff et al. (2017) used unique flanking sequences acting as a barcode in a pool of collected constructs that can be retrieved using PCR and subsequently assembled using Golden gate assembly. Biopart Assembly Standard for Idempotent Cloning (BASIC) assembly exploits orthogonal linkers to avoid PCR entirely and achieved over 90% accuracy with a 7-part reaction. Devised a hybrid method, Golden Gate-Gibson (3G) combines overhang assembly with Golden Gate style part libraries (Halleran et al. 2018). Despite the relatively rapid speed of modern assembly methods, construction of large libraries of clones can still take a significant amount of time. It is here that automated construction methods using robotics or microfluidics (discussed later), would greatly aid in speeding up the process as they can potentially run many assemblies at once 24 h a day with minimal human supervision.

## Test: high-throughput circuit characterization

After the ‘build’ part of the cycle, the next part is to ‘test’; quantifying the characteristics and dynamics of the circuit to inform on future designs and find solutions to any failures that have been encountered (debugging). Scalability in characterization is largely determined by the feasibility of running many concurrent experiments at once, measuring many different properties and gathering precise data from the samples reducing the time needed to complete the DBTL cycle.

Traditionally, this has been dominated by fluorescent gene reporters such as green fluorescent protein (GFP) and red fluorescent protein (RFP). These proteins are used to measure gene expression and quantified using a plate reader or a fluorescent microscope. Beyond measurement of the regulatory sequences of gene expression, they can be fused to other proteins to study protein localization and interaction through Förster resonance energy transfer (FRET) (Selvin 2000). The advent of flow cytometry has enabled the simultaneous measurement of multiple cellular properties in every cell, such as size, granularity and fluorescence analysis through multiple lasers fed by a small current of cells suspended in fluid. Flow cytometers can even separate cells by fluorescence levels known as fluorescent activated cell sorting (FACS) (Tracy et al. 2010). This analysis of single cell data offers a much more precise view of cell state and reveals a much deeper relationship between host-circuit physiology, such as the relationship between fluorescence and cell volume, something that is much more difficult in population level measurements.

Measurement of the fluorescence of a protein encoded downstream of a regulatory sequence is equivalent of measuring the final product of gene expression. This expression measurement combines both transcription and translation together and doesn’t differentiate between the two. Pothoulakis et al. (2014) developed the ‘spinach’ RNA aptamer which fluoresces in the presence of the fluorophore, 3,5-difluoro-4-hydroxybenzylidene imidazolinone (DFHBI), and is consequently a good option to measure transcription separately. Another limitation is the incorporation of undesirable genetic context (discussed in more detail later on) such as variable untranslated regions (UTR) of DNA that can form secondary structures disrupting translation though themselves not being translated, this can be addressed through the use of insulators that reduce context effects such ribozymes that cleave the 5′ UTR (Lou et al. 2012). The expression of these protein can also cause variable metabolic load (Bentley et al. 1990), making it hard to measure the expression of many genes in parallel and minor variations in experimental conditions can cause large changes in expression (Kelly et al. 2009; Rudge et al. 2016). To solve this issue, Kelly et al. (2009) normalized promoter activity using a reference promoter resulting in the relative promoter unit (RPU), and Rudge et al. (2016) compared the output of reporter genes concurrently with a control plasmid to find the intrinsic promoter activity, reducing the variance due to extrinsic factors to less than 4%.

High throughput experiments allow us to repeat many experiments in parallel that gather a greater quantity of data in a shorter space of time whilst genome and proteome wide techniques offer a wider view of cell state that singular gene expression experiments cannot practically offer. For example, using RNA-seq we can get a non-invasive snapshot measurement of the RNA levels of single cells and populations, enabling complete analysis of changes when implementing genetic circuits. Based on next generation sequencing, mRNA is cut into small sections and turned into complementary DNA (cDNA) through reverse transcription before sequencing and alignment. RNA-seq has been used to analyze the transcriptome of cells and has been demonstrated to work in situ in mammalian tissues (Lee et al. 2014). Liao et al. (2017) used RNA-seq to determine changes in the host cell transcriptome when an AND-gate circuit is designed under different circuit compositions and in different plasmid copy numbers; finding that higher copy number decreased the orthogonality between the circuit and host gene expression in addition to increasing metabolic load and causing imbalance among the circuit components. Gorochowski et al. (2017) used RNA-seq to measure simultaneously part performance and the state of a three-input one-output circuit comparing 46 parts. They were able to debug failures in the circuit due to antisense promoters, terminator malfunction and media related failure and make informed design decisions, such as including a bi-directional terminator to cease antisense transcription. Limitations in terms of cost and library preparation time are being addressed with simplifying techniques such as RNAtag-seq whereby DNA barcodes are uniquely tagged to allow early pooling of samples before the preparation of the library (Shishkin et al. 2015). Other methods include single molecule RNA fluorescence in situ hybridisation (smFISH), used by Nielsen et al. (2016) to quantify mRNA levels of yellow fluorescent protein (YFP). 25 oligonucleotide probes, each 20 bases in length were fluorescently labelled with TAMRA (carboxytetramethylrhodamine), and binding of multiple probes enables sufficient fluorescence to detect and localize target mRNA. Similarly whole cell mass spectrometry can be used to attempt to identify changes in the proteome (Ouedraogo et al. 2013).

Prototyping gives meaningful biological information towards the design of the final system yet can be assembled and tested much more quickly. Cell free in vitro systems have all the machinery necessary for basic protein expression but do not require long culturing times, often also omitting the complexity of full host metabolism and thereby being significantly easier to model. They also offer options to monitor dynamics of the system in real time with fluorescent RNA aptamers (Niederholtmeyer et al. 2013) and FRET probes (Norred et al. 2015), also facilitating direct reaction sampling into a HPLC or MS machine (Heinemann et al. 2017). Drawbacks include being much more lacking in some shared cellular resources (Gyorgy and Murray 2016), as well as having potential energy consumption imbalances (Garamella et al. 2016). One example is the *E. coli* transcription-translation based cell free system (TX-TL) which has successfully been used for prototyping promoters (Iyer et al. 2013) and negative feedback loops (Karig et al. 2012). Further advances enabled use of linear DNA through protection from degradation by RecBCD through the addition of GamS protein (Sun et al. 2014). Using this method a 4-piece genetic switch was assembled within 8 h (1 working day), using simple Golden Gate assembly and polymerase chain reaction (PCR) to create 4 linear sequences directly used for testing, although in this case there was a lack of correlation between in vivo and in vitro results (Sun et al. 2014). Another example built on the concept of using whole cell extracts by using microbial consortia to obtain purified translational machinery (Villarreal et al. 2017). Pardee et al. (2014) demonstrated freeze dried paper based cell free systems containing gene networks that can be rehydrated when needed.

A different way to increase scale is to minimize the resource consumption of each experiment and to automate physical tasks. The field of microfluidics deals with the precise manipulation of small amounts of fluids in the micro and nanoliter scale. Discrete volumes of liquid can be packaged into droplets and controlled automatically either as a solution or individually, each droplet functioning as an independent reaction mix with the small scale enabling conserved use of reagents and biological material. Typically these methods are either continuous; using oil and water to generate a controllable liquid stream, or digital; using voltage to control the movement of individual droplets on a conductive material (Huang and Densmore 2014). Shih et al. (2015) demonstrated that it was possible to concurrently run several assemblies at once (Golden Gate, Gibson and yeast), using a hybrid of both microfluidic technologies, assembling a library of 16 plasmids and performing on chip electroporation into bacteria and yeast. Other systems demonstrated heat shock transformation and the capability to culture the cells on chip (Gach et al. 2016). Analogous to FACS, Baret et al. (2009) sorted cells by fluorescence through fluorescent activated droplet sorting (FADS). Procedures have been developed that can trap single cells in droplets, and provide them with all the nutrients needed to be incubated for days, permitting longer study and performance that cannot be obtained through FACS (Bennett and Hasty 2009).

Uniting cell free and microfluidic technologies can combine the benefits of both, for example, generating many artificial cell-free entities, prototyping in parallel. By example, Schwarz-Schilling et al. (2016) produced functional AND gates and sender circuits in droplets containing cell free systems and bacteria. Fan et al. (2017) use droplet microfluidics to print accurate and small quantities of cell free systems to measure interactions between three genetic factors at a synthetic promoter and used this data generate a model. Wang et al. (2018) used a similar method, only combining a locked nucleic acid probe (measuring mRNA levels) with fluorescent proteins enabling simultaneous measurement of transcription and translation in massively parallel cell free droplet experiments.

## Learn: biological circuit design automation and modelling

The synthetic biology community has developed software tools that aim to replicate the success of computer aided design (CAD) used in electronic circuit engineering. Design automation has the potential to accelerate biological design by allowing designers to access existing knowledge of biological parts, arrange parts into circuits, design experiments, store and visualise experimental data, and potentially make predictions about circuit behaviours. For circuit construction (build), it could plan out assembly of the physical DNA sequence from the given starting material and include the experimental protocol needed to do so. For testing, software might enable experiments to be designed and simulate a computational model of the system, allowing costly and time-consuming experiments to be replaced, but still give insight into how a system might behave and identify which experiments are critical or contain the most information for guiding design decisions.

Several software tools have emerged over the last 10 years that seek to deliver some of these features, including Genocad (Czar et al. 2009), CellDesigner (Funahashi et al. 2008), Biojade (Goler et al. 2008), SynbioSS (Hill et al. 2008), Tinkercell (Chandran et al. 2009), Visual GEC (Pedersen and Phillips 2009) and Cello (Nielsen et al. 2016), although many of the former projects are dormant. Some software forgoes biological part data and only operates at the abstracted design level (Bhatia et al. 2017), whilst others are primarily concerned with data storage standards, such as DICOM-SB (Sainz De Murieta et al. 2016), many instances of software use the Synthetic Biology Open Language (SBOL), an open standard for the representation of genetic parts, with common formats for both data and visual symbols (Roehner et al. 2016). Most also offer the export of the models via the Systems Biology Markup Language (SBML) (Finney and Hucka 2003; Hucka et al. 2003), which enable model analysis in more general software platforms, such as Matlab and Copasi.

Cello, one of the latest iterations of gene circuit design software enables circuits to be constructed that compute specified logic functions. The Verilog logic programming language is used to describe circuit function, and a user constraints file to specify parts and organisms to create a searchable design space. Circuits are modified to be compatible with a library containing NOR and NOT gates based on repression. The system is simulated to predict circuit performance, factoring in load, population variability, growth, and connectivity in terms of RNA polymerase (RNAP) flux. Finally, the physical component is designed for assembly, i.e. the circuit contained in one plasmid and the reporter on another with appropriate promoters, terminators and other gene regulatory elements on each (Nielsen et al. 2016).

**Fig. 4:**
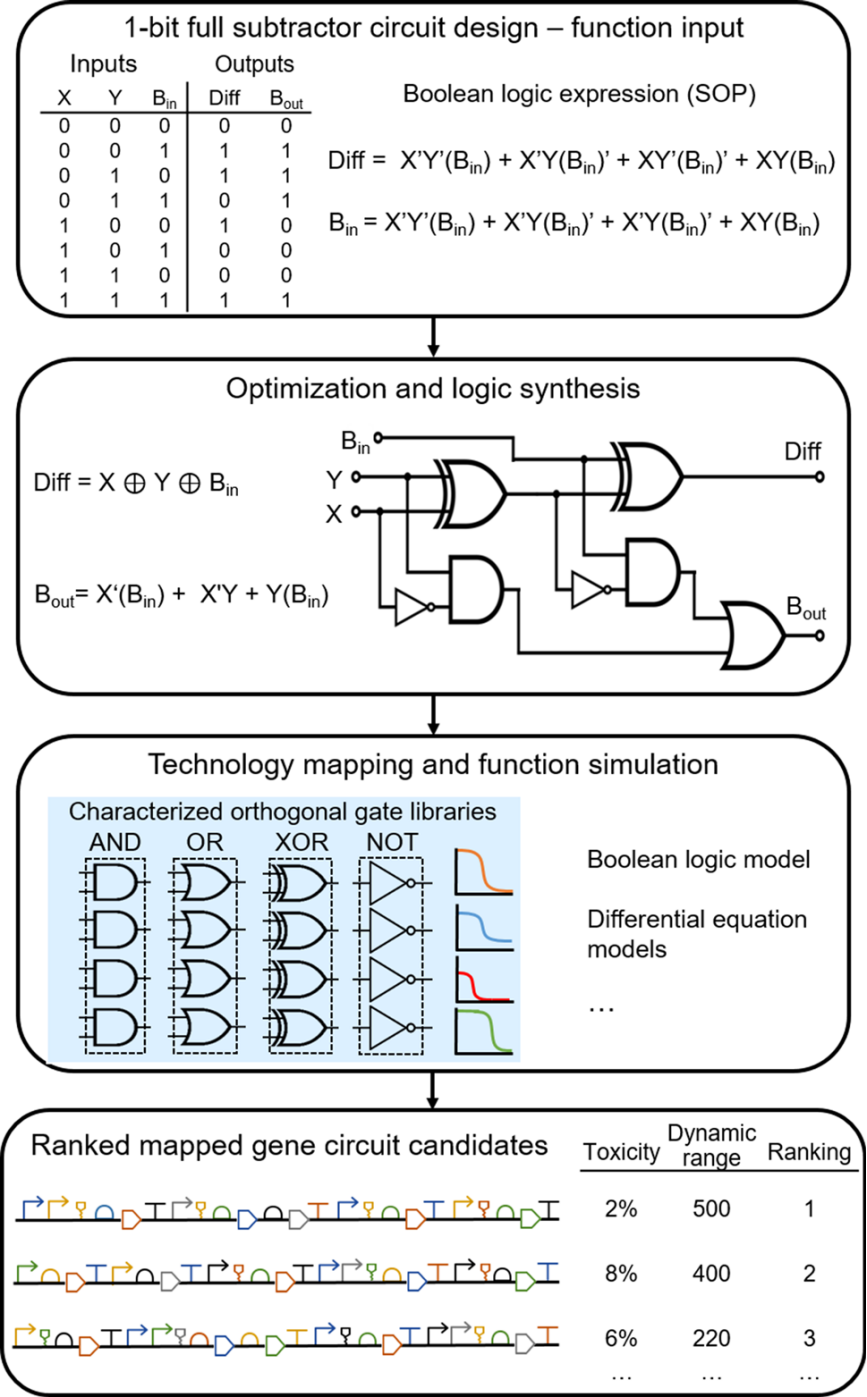
Towards large-scale genetic circuit design automation. Representative design flow of an exemplar digital logic gene circuit is shown. Design input in an abstracted format, in this case a truth table and a Boolean logic expression (SOP = sum of products). The system then performs an optimization in Boolean algebra for the 1-bit full subtractor. Logic synthesis is performed to design the most efficient circuit using a set of preferred types of logic gates; parts and devices are assigned from a characterized gate library (mapping) and modelled to assess feasibility of implementation. The genetic sequences are deconstructed and reconstituted into an optimal sequence design to be used in the target host organism. These are then ranked using important factors when implementing in vivo. Examples used in this case are toxicity effects on the host in terms of percentage growth reduction and dynamic range of the output for the circuit, measured in fold change between the ON and the OFF states

Figure 4 shows a representative gene circuit design automation flow, inspired by electronic circuit design automation, for an exemplar 3-input and 2-output 1-bit full subtractor that comprises 7 logic gates wired in 5 layers. Initially there would be an abstract level of input of the circuit function, such as using a graphical user interface (GUI,) a truth table, or Boolean algebra, before an optimization of the overall design to reduce the number of parts to a minimum whilst retaining the desired function. Subsequently, logic synthesis would be performed to transform the expression into desired gate level format before technology mapping all the possible circuits from standard well characterized libraries of logic gates and parts, such as the open iGEM parts registry (Mitchell et al. 2011) or SynBioLGDB (Wang et al. 2015b). Potential systems would be modelled for functionality and ranked accordingly, and genetic assembly constructs would be designed for assembly.

One of the major challenges for design automation software is the difficulty in producing mathematical models that are predictive of circuit function. This is challenging for several reasons. The first major challenge is that it is difficult to know how to even write down a mathematical model that captures the nonlinear features of the biochemical interactions of a given circuit. To model a system mechanistically (as opposed to purely statistical models), the main elements of the system should be known and their interactions with the genetic and protein elements of the cell specified directly (the intricacy of which depends on the scope of the model). This requires either prior knowledge or the mining of interaction databases, though these are far from exhaustive at present. There is also the consideration of chemical diffusion and spatial arrangement (Endler et al. 2009). Subsequently mathematical formulae can be determined to describe the system. Beyond intrinsic circuit interactions there are also interactions between circuit components and host resources (which we return to later in this review).

Another challenge relates to the fact that chemical reactions are discrete and stochastic, although modelling these variations can be computationally burdensome. In some cases, deterministic approximations can be sufficient depending on the scope of the answer desired. Simpler still, Boolean approximations can be very effective for describing gene networks where the gene is notionally simply on or off, and when intermediate expression is not functionally relevant. Models could be based on, amongst others, ordinary differential equations (ODEs), partial differential equations (PDEs), stochastic differential equations (SDEs), reaction–diffusion equations, and either stochastic sampling or integration of the chemical master equations (CME) (Chandran et al. 2008; Marchisio and Stelling 2009; MacDonald et al. 2011). How to assess which model structures are most appropriate (multimodel selection) is a problem known to be extremely challenging, even when it is possible to perturb and directly measure many of the circuit components (Marbach et al. 2010; Hayden et al. 2016). Nevertheless, approximate Bayesian methods are gaining traction for deciding between a set of similar models (Toni et al. 2009).

A related (simpler) problem to model selection that is still of practical utility is the problem of inferring model parameters (of a fixed model structure), given observation data (Toni et al. 2009; Golightly and Wilkinson 2011). After all, to make accurate predictions about circuit behaviour, the parameter values must surely be known to some level of accuracy. However, the majority of biological components described thus far in this review have not been characterized in sufficient detail to enable predictive modelling to be commonplace in synthetic biology. With recent advances in machine learning, there are now methods emerging that can handle high-dimensional parameter inference problems, though require the use of large-scale computing resources. Nevertheless, such methods enable us to determine the relationship between the sequence of DNA parts (Alipanahi et al. 2015; Kreimer et al. 2017), and their quantitative behaviour in the cell, including the RBS calculator described above (Salis et al. 2009), but also more generally how protein expression depends on the whole 5′ untranslated region (Cuperus et al. 2017). But all circuits also incorporate other modes of biochemical regulation (e.g. protein turnover, ligand binding, translocation), which means that methods for inferring parameters for models of specific circuits is just as important. Characterizing sets of related circuits simultaneously is beginning to enable model parameterizations from no prior quantitative information directly from measurements, enabling design optimisations to be generated (Huynh and Tagkopoulos 2014, 2016; Grant et al. 2016).

## Towards advanced paradigms in cellular computing

### Memory and data storage

Memory in cells relies on permanent cellular changes in response to temporary inputs, normally genetic, this enables sequential logic over common combinatorial logic. One of the earliest reported devices was the bistable toggle switch, containing two stable gene expression states (Gardner et al. 2000). Subsequent work used recombinases, enzymes that can catalyze DNA excision or flipping depending on the direction of the corresponding attachment (att) sites that flank the DNA sequence of interest (Bonnet et al. 2012). Some recombinases are bidirectional, either inherently or alongside a co-expressed Recombinase Directionality Factor (RDF). However, often permanent reactions are preferred thanks to their inherent stability. Reading the output can be as easy as basic sequencing. Alternatively, there is a possibility of enclosing parts such as promoters and gene coding sequences which can be flipped in and out of the correct gene coding orientation enabling circuit integration for sequential logic. Siuti et al. (2013) were able to create 16 two-input Boolean functions using recombinases that surrounded genetic elements such as promoters and terminators, demonstrating memory stable for 90 cell generations and Yang et al. (2014) reached a recording capacity of 1 byte.

State machines can be one of a number of finite states at any given time, with access to states dependent on predetermined sequence of events triggered by various conditions. A basic 3-state version is shown in Fig. 2c, and the most complex reported has 16 different positions (Roquet et al. 2016). A version of this machine was used to record temporal events in a population, with the distribution of final cell states and spatial location recording the dynamics of any inducer response including pulses (Hsiao et al. 2016). Another example created analog-like memory by generating graded expression of single stranded DNA in response to various signals, co-expressed with a corresponding recombinase targeting specific genomic sites resulting in a mutational response in the genome proportional to the duration and magnitude of the input (Farzadfard and Lu 2014). As mentioned in the introduction, Weinberg et al. (2017) used recombinases to build over 100 different types of multi-input multi-output circuits that include a 1-bit full adder, by taking advantage of recombinases they were able to combine computation into a single layer.

The natural CRISPR (Clustered Regularly Interspaced Short Palindromic Repeats) and CRISPR associated protein (Cas) system stores DNA from invading species in the genome to generate an immune response. Memory can also be stored by co-opting this approach as shown in Fig. 2d. Recoverable via sequencing, Shipman et al. (2017) assigned colours to the sequences and by distributing the system amongst many cells, they were able to construct a simple image recorder, with a capacity of 2.6 kilobytes. Akin to this method, another group recorded the occurrence of certain metabolites by initiating production of trigger DNA in their presence (Sheth et al. 2017), others modified the sequence of recorder plasmids, shifting and measuring the ratio between modified and unmodified plasmids (Tang and Liu 2018). Another recent example used DNA methylation to record events; repressed by a zinc finger in the off state, induction by heat methylates the DNA and prevents binding thereby activating permanent expression (Maier et al. 2017).

### Distributed computing

Most of the circuits have thus far been localized in a single cell. However natural systems can organize around different cells by using intercellular communication. Analogously much work has also gone into spatially distributing gene circuits across multiple cells, replacing the intracellular ‘wires’ with synthetic intercellular signalling pathways as seen in Fig. 2e. This allows components to be reused whilst avoiding any potential ‘cross talk’, enabling scale up of vastly larger systems from a much smaller library of parts (Macía et al. 2012) as combining the outputs of separate gates can functionally recreate the logic behaviour of a single more complex one (Regot et al. 2011; Macía et al. 2012).

In bacteria, signal propagation between cells can be achieved by adapting the quorum sensing molecules, *n*-acyl homoserine lactones (AHLs), natural molecules secreted by cells that allows for coordinated activity such as biofilm formation based on cell density (Tamsir et al. 2011). Early circuits were small (Regot et al. 2011) but recent ones are much larger, such as the 6-input multiplexer (Macia et al. 2016) and the most complex system to date, a 1-bit full binary adder; incorporating 22 separate gates distributed amongst 9 specialized mammalian cell types in a complex three dimensional environment (Ausländer et al. 2017). Guiziou et al. (2018) created an automated design framework that did not require cell to cell communication using integrase networks distributed across multiple cells. Memory, like other functions, can also be distributed into different cells, the bistable toggle switch was effectively replicated by two cells containing a NOT logic gate, each gate repressing the other when activated, communicating through inter-cellular signalling (Urrios et al. 2016). This level of distributed computing can be applicable to metabolic engineering for synthetic product production where different populations each handle a different part of the pathway in a division of labour (Tsoi et al. 2018). Other potential applications include using cell–cell signalling to induce structural self-organisation of tissues (Toda et al. 2018) and pattern formation (Basu et al. 2005; Karig et al. 2018).

## Challenges and outlooks

There are number of challenges which become especially relevant when scaling up the size and complexity of gene circuits for useful functions. Namely the ability of a circuit to sense inputs and generate useful outputs, manage resource consumption, and maintain modularity of parts. In particular, modularity faces challenges in orthogonality, retroactivity and avoiding undesirable behaviour arising from genetic and cellular context. Each problem will be defined, its importance explained, the current state of the art and future prospects examined.

### Modular, robust and well characterized parts

Generally, larger circuits contain more parts with each part and connection representing another point of failure. Therefore parts must be well characterized with robust and predictable behaviour regardless of context, enabling the design of large-scale circuits to be fast, predictable and reliable. Essentially this refers to modularity, whereby parts retain their inherent function and behavioural characteristics irrespective of the conditions that they are placed in Sauro (2008). This enables two key processes to occur, the decomposition of a system into individual parts which can be constructed and tested separately and the subsequent construction of larger systems and from a library of smaller well understood pieces which generate predictable functions. Modularity is difficult because of several overlapping yet distinct challenges common to biological systems: connectivity, retroactivity, orthogonality, and context effects.

Connectivity in this scenario refers to the ability of parts to communicate reliably with other parts, robust signal propagation down a system is important to generate a consistent output, if a signal degrades due to noise or is unable to be propagated it can disrupt function. Therefore it is desirable to protect circuits by maintaining good connectivity.

Context dependency is the phenomena whereby part behaviour becomes dependent or affected by unwanted interactions from the host, environment, or even its own composition (Cardinale and Arkin 2012). Unlike in electronic circuit design modules are linked by discrete wires, which when layered correctly are unidirectional and do not propagate signals to unintended recipients and have minimal interaction with the surrounding environment. This trait is increasingly important as circuits get larger and more complex, as cross talk leads to noise and unpredictability. Functionality can break down due to these unwanted interactions with the host, system and environment (Kwok 2010; Wang et al. 2011; Wang and Buck 2012; Liu et al. 2018a, b). A part that does not interact significantly with this context can be surmised to be orthogonal (Liu et al. 2018a). Orthogonality therefore, is important for both functionality and modularity.

The problem of context runs deep, even genetically identical cells in the same environment can show variable phenotypes; attributed in part to stochastic gene expression due to the variable nature of small numbers of interacting molecules (Munsky et al. 2012). Synthetic pathways can elicit responses in the host such as stress or simply display toxicity and circuit performance is tied closely to the health of the host, its physiology, the growth rate, and (discussed later) the availability of resources both internal and external, cell volume and even division state (Cardinale and Arkin 2012; Brophy and Voigt 2014; Liao et al. 2017). Context can also extend into environmental factors such as pH or media (Wang et al. 2011). Temperature in particular has been shown to affect the rate of transcription and the secondary structure of DNA and RNA (Cardinale and Arkin 2012). There is also genetic context; expression can be disturbed by the composition of the adjacent DNA sequences resulting in UTRs affecting the secondary structure and translation rate of the mRNA (Reeve et al. 2014). The size and copy number of the host plasmid can also affect behaviour (Liu et al. 2018b). DNA folding and spacing can affect the steric (spatial) ability for transcription factors to bind, sequence homology can cause deleterious effects and even the orientation of genes on the plasmid can modulate expression levels (Yeung et al. 2017). Since replication of the DNA must occur, there will be errors and possible rendering of parts non-functional. Because many parts have a negative effect on cell health and growth, eventually populations will incorporate an increasingly large subsection of non-functional circuits, this is known as genetic instability (Zhang et al. 2016). This is in spite of selection methods with, for example, antibiotics as the cells will still evolve to only retain the minimum number of genes required.

Retroactivity specifically, was defined by Jayanthi et al. (2013) as “the phenomenon by which a downstream system changes the dynamic state of an upstream system in the process of receiving information from the latter”. In this case, downstream and upstream are relative to the intended flow of information (Del Vecchio et al. 2008). Essentially, this means that attaching example part B to receive the output of example part A will change the way part A behaves, this of course scales as a problem the more connections there are. In biology this can occur when upstream regulatory factors bind their downstream targets. This gets worse as the ‘load’ increases (the number of sites relative to factors), and is magnified in larger circuits, as when signalling molecules are bound they can no longer transfer information.

The question of context, orthogonality, signal strength and modularity, although distinct are also overlapping challenges and have interacting solutions. One of the best ways to maintain a robust signal in digital like circuits is to maintain a large dynamic range, that is, a large difference or ratio between the ON and OFF state. Although by nature analog graded responses are more vulnerable to noise as they have continuous outputs, this remains true for both as a large dynamic range means the relative effect of any noise is smaller providing the scale of noise remains the same. This protects the signal from degradation as it propagates throughout a system, which itself lends itself to modularity, as a strong signal can mean behaviour remains robust throughout different environments. The tuning tools discussed earlier in Sect. 2 are the often used in adjusting response curves and in optimisation to ensure the output of one part can be received and function as a relevant input for the downstream part. They can also affect the dynamic range as well as modulate retroactivity by increasing expression of the component, (Brophy and Voigt 2014). Alternatively, signal strength can be modulated by the addition of other parts such as amplifiers (Wang et al. 2014).

Other solutions to retroactivity have been attempted by borrowing of concepts from control theory and the subsequent addition of feedback and feed forward loops for insulation, although the latter can only be used based on how the disturbance affects the system, thereby being a much more specialized solution (Del Vecchio et al. 2016). The ideal insulator has zero retroactivity to the input and is not functionally affected in terms of output after taking on the load. One possibility is to use phosphorylation-dephosphorylation cycles since they work on a much faster timescale and do not place a large metabolic burden on the host (Del Vecchio et al. 2008).

To avoid crosstalk within a circuit, we must minimize unwanted interaction with the host and other sections of the circuit. This generally means avoiding repeat use of the same parts, in turn requiring proportionally more parts to increase the complexity and scale of a system making the expansion of the library of well characterized orthogonal parts essential. Alternatively, the circuit can be insulated from unwanted interactions, for example, the circuit can be constructed as to not rely on the host transcriptional machinery (Liu et al. 2018a) or follow the multicellular distributed approach mentioned previously. The former has gained some traction within the community. The phagemid T7 RNAP has been co-opted to separate the transcriptional machinery from the bacterial host Temme et al. (2012). Chan et al. (2005), refactored the T7 RNAP itself by isolating genes through physical separation, removing or standardizing adjacent sequences to the coding region whilst retaining functionality, making it much simpler to model and easier to manipulate. This has further led to the idea of an entirely orthogonal central dogma, conceptualising the addition of orthogonal DNA polymerases, aminoacyl-tRNA synthetases, and ribosomes for replication and translation respectively, (Liu et al. 2018a). Cello has incorporated into its design space strong terminators preventing RNAP read-through and ribozyme binding sequences and secondary structures that can cleave off the UTR to standardize context (Lou et al. 2012; Nielsen et al. 2016). Carr et al. (2017) developed a degenerate insulator screening (DIS) technique to determine exact levels of insulation desired for bacterial promoters. Lengthy DNA sequences can be compressed by sharing regulatory parts, though paradoxically this will take it out of the genetic context it was characterized in, adding more uncertainty (Brophy and Voigt 2014). Lowering expression of and reducing resource consumption as well as reducing the number of repeated sequences and using inducible promoters can provide a reduction in genetic instability, as well as tying the function of the circuit to host health thereby making it advantageous to host survival (Sleight et al. 2010). Noise can be resisted with negative feedback, as well as from feed forward loops which incorporate both positive and negative regulation, whilst cell–cell communication has also been suggested to have robustness to noise (Zhang et al. 2016).

Understandably, for large scale gene circuits, all of these issues are proportionally magnified. The more parts the more points of failure. Ideally, there would be a large number of highly modular components that could easily be assembled together with predictable behaviour, in a sense ‘plug in and play’. However this is far from the case: despite the wide array of parts that have been described in the literature many of them are not well characterized enough to facilitate easy reuse. Simply put, the behaviour of many components becomes less predictable as they taken are taken further away from their original context. This is partly down to a lack of standardization in characterization. Protocols vary across groups, equipment also differs and characterization will be subject to a host of specific design factors such as plasmid, strain and reporter choice which often we do not understand well enough to reliably predict behaviour when they are changed. The latter problem is a result of our general lack of knowledge regarding basic biological system behaviour. Whilst mapping out potential cross reactivity between a small libraries of parts is reasonably feasible, mapping all potential connections and determining all possible interactions with the host is an order of magnitude more complex and can be even higher if accounting for changing environments and different species across time and space. Not only would this be computationally burdensome and difficult to mathematically model, it would also require a heavy amount of accurate and precise data that simply does not exist in the required scale.

Although optimisation steps listed above are possible, the time cost of performing optimization steps in multiple components is vast and any cross talk only increases the time needed as parts respond to multiple unwanted factors and become more difficult to adjust. In a recent pressure test where organisms were to be engineered to produce 10 molecules unknown in advance, Casini et al. (2018) noticed that literature searches and database entries did not produce actionable data,and even standard procedures such as sequence verification and plasmid/oligo design became bottlenecks. In addition, they had to wait 3-8 weeks for DNA synthesis further reducing available bench time suggesting that there is room for improvement across the board.

The solution to these problems will lie in more accurate and standardized initial characterization of parts, improved understanding of basic circuit–circuit and circuit-host interactions to predict behaviour under different conditions and the reduction of man-hours required using high throughput automated design, construction and characterization methods. It is in this context that large scale circuits could benefit from the scale up and automation of microfluidics for tasks such as genetic assembly and high throughput characterization experiments that gather precise single cell data (which offers a much deeper understanding of host-circuit physiology than population averages), cell free systems enabling rapid prototyping, and methods such as RNA-seq giving us a much wider view of cell state. The resulting data can then be fed into computational simulations and models in order to be fed into the next round of the DBTL cycle. This will result in a positive feedback loop of knowledge; as circuits become better characterized, our understanding of systems will increase, further informing our design, our ability to model and predict behaviour and subsequently reducing the time needed to complete the DBTL cycle.

### Generating relevant inputs and outputs

For circuits to have pertinent real-world applications, they must be able to sense relevant phenomena such as the intracellular concentration of a metabolite or extracellular factors such as heavy metals, RNA, DNA, protein, pH, light, oxygen or heat. In addition they must actuate outputs that are valuable to human endeavour. By doing so gene circuits can make the leap from interesting academic problems to useful biotechnological applications.

The generation of novel functional parts often finds its inspiration in already existing natural systems, although a degree of characterization and refining of these parts is necessary to add them to the toolbox (Wang et al. 2015a). Existing proteins have been engineered to sense new metabolites through directed evolution (Collins et al. 2006; Taylor et al. 2016) and some hybrids with novel function have also been developed. A synthetic light-sensitive sensor kinase (Cph1–EnvZ) was made in *E. coli* by fusing the photoreceptor domain of the phytochrome Cph1 protein from Synechocystis to the intracellular signal transduction domain of the *E. coli* EnvZ kinase, yielding a functional sensor chimera (Tabor et al. 2009). Antibody domains have been fused with DNA binding domains and activated via ligand induced dimerization to enable sensing of new molecules (Chang et al. 2018) and chimeric custom proteins have also been demonstrated with modified Notch receptors (Morsut et al. 2016). In some cases sensors can be modified to work in different hosts, as demonstrated with the retooling of TetR family repressors, to work in human embryonic kidney (HEK293) and Chinese hamster ovary (CHO) cells (Stanton et al. 2014). Examples of outputs include useful biological or small molecule products (Paddon and Keasling 2014), simple signalling responses to difficult to detect stimuli (Wang et al. 2013a; Bereza-Malcolm et al. 2015), to the cancer targeting classifier circuits that secrete apoptotic proteins (Xie et al. 2011).

Larger scale circuits will likely include a greater number of these unique sensing and output parts that will enable complex programmable functionality. For example, a bioremediation based system could potentially monitor many environmental inputs and secrete specific enzymes that degrade waste products in response. Circuits would benefit then, from a larger library of unique well characterized and modular parts, the general challenges and solutions of which have already been discussed. In particular, the ability to link novel inputs and outputs would benefit strongly from improved protein engineering techniques in modifying existing functionality or the building of chimeric proteins. In turn this would strongly benefit from deep structure function understanding to avoid time consuming trial and error experimentation (Wang et al. 2013b). Bioinformatics may be able to play a strong role too, in estimating structure and function of candidate proteins from their genetic sequences to narrow the design space (Stanton et al. 2013).

### Metabolic burden

Metabolic burden or load can be understood as the resource consumption required by the engineered system upon the host. The concerns of burden are often the focus of metabolic engineers when optimizing a product producing pathway, however it is also relevant in the construction of gene circuits as resource limitation fundamentally affects system behaviour. Cells have an upper limit of nutrient and energy intake that limits all cellular activity, one of these hard limits can usually be described in terms of ATP. Cells can compensate somewhat by increasing respiration and catabolism but under too much strain there is a sharp drop in total protein production to near 0 and often results in the collapse of the population (Wu et al. 2016). The effect of foreign protein production on the host was spotted early on; increasing amounts of foreign protein production led to decreasing growth rate in *E. coli* (Bentley et al. 1990; Bhattacharya and Dubey 1995). The amino acid content of recombinant proteins has also been shown to affect production levels (Bonomo and Gill 2005) whilst the amount of free ribosomes and RNAPs is also important, itself affected by presence of plasmid DNA (Birnbaum and Bailey 1991). There is evidence that genetic load resembles the equations of Ohm’s law for resistance in electrical circuits (Carbonell-Ballestero et al. 2016). Other findings have shown that ‘leaky’ basal levels of transcription and high plasmid copy number contribute to the protein burden (Lee et al. 2016), with copy number also changing gene circuit expression as well as in the host cell. Increasing copy number increases expression of the receptors to the system input, thereby increasing retroactivity, decreasing the sensitivity and dynamic range of repressor based systems given the same amount of repressor, and vice versa for activator based systems (Wang et al. 2015a; Liu et al. 2018b).

Managing load requires accurate characterization and calculated mitigation. The copy number and general expression levels of the circuit should be as low as is essential for predictable behaviour. If necessary, the circuit can be spread into multiple cells following the principles of distributed computing. RNA based control tends to be the least burdensome on host metabolism; Lapique and Benenson (2017) even combined two orthogonal binding sites into one DNA sequence using recombinases to reversibly express equal amounts of the forward and reverse DNA sequence, thereby generating two separate species of RNA, each with one functional and orthogonal binding site. Ceroni et al. (2015) inserted a constitutively expressed GFP element that would act as a tracker for metabolic change in the host. The Cello design framework manages burden through simulating the load on each cell by factoring in the impact on growth relative to the functional activity of the input promoter (Nielsen et al. 2016). This information can be used by the designer to optimize the circuit (Wu et al. 2016). Liao et al. (2017) created a model that considers different RNA levels, the proteome (dividing it into gene expression apparatus and metabolic machinery), resource partitioning (including ATP and amino acid synthesis) as well as other factors such as growth, copy number and cell volume. The CRISPR-Cas system has been used to attenuate leaky gene expression with T7 RNAP and has been shown to improve growth in systems with previously toxic leaky expression (McCutcheon et al. 2018). Incoherent feedforwards loops (iFFL) have been engineered into promoters using transcription-activator-like effectors (TALEs) which stabilised expression level at different copy numbers (Segall-Shapiro et al. 2018) whilst Lee et al. (2016) created single copy plasmids with stable expression.

Larger circuits mean more components and this will inevitably have a proportionally larger effect on metabolic load. Selecting parts that have minimal resource consumption (such as RNA based tools), and reducing consumption of existing through tuning will constitute a large part of the solution. In the latter case, there are complications as once a part is modified away from its original specifications, it will need to be characterized again. Furthermore, reducing the expression level can have negative effects on signal robustness and increase the susceptibility towards unwanted interactions and noise. The literature has suggested that parts with analog behaviour are significantly more resource efficient and the authors suggest that hybrid devices will likely be common in the future (Sarpeshkar 2014). Parts might be also arranged so as not to overlap on the type of load they produce, for example, distributing load across both transcription and translation, or they might be combined into a single layer that does not require communication between parts for sub-computation as demonstrated earlier (Weinberg et al. 2017). However the authors do note that this means the performance of circuits cannot be predicted based off its constituent parts. Another solution would be to distribute the circuit into different consortia, as discussed earlier; likely to become a common approach as reduction of load from individual parts cannot decrease indefinitely.

Tools that allow us to monitor and predict load will also become increasingly important. Here the related field of metabolic engineering may hold some promise. High throughput experimentation again will allow us to gather a larger amount of data in a shorter space of time and tools such RNA-seq or whole cell mass spectrometry that offer a wide view of cellular gene expression and metabolism to be key in deciphering the interactions between circuit and host (Liu et al. 2018b). Here the related field of metabolic engineering holds promise, having developed tools such as metabolic flux balance analysis to predict the distribution of important resources such as carbon (Yang et al. 2007). Finally, like as before, as data becomes more readily available and accurate, computational prediction will become increasingly important.

## Concluding remarks

Gene circuits hold great potential for addressing real-world challenges including applications in biomanufacturing (Si and Zhao 2016), biosensing (Bernard and Wang 2017) and biotherapy (Riglar et al. 2017). Larger scale systems potentially enable more intricate control and the larger circuits thus far discussed have been able to compute more complex functions than the smaller ones. Circuits have been steadily increasing in size, albeit slowly, and the molecular toolbox available to synthetic biologists is now larger than ever before. There has been a significant expansion of orthogonal parts that enable a vast quantity of versatile methods to control behaviour, providing a solid foundation for constructing complex circuits.

However there remains a significant lack of predictability of the behaviour of parts when put together that scales in larger systems preventing regular reuse of all but the most basic parts. Modularity and standardization remain issues for biological components and there are fundamental gaps in our knowledge on basic biological processes that prevent us from accurately predicting changes. Recent advances in characterization techniques enable high throughput experiments providing single cell and genome or proteome levels of data, whilst new methods in microfluidics and cell free systems potentially allow for high speed prototyping of systems in a matter of hours and days instead of weeks. The increase in time efficiency in the laboratory whilst simultaneously gathering larger data sets promises a positive feedback loop that enables increasingly faster iterations of the DBTL cycle that concurrently will result in larger more robust systems as well as a leap in our fundamental understanding of biological interactions. Automated systems can already be seen in industry at the start-up stage, at companies such as Ginkgo bioworks and Zymergen (Anne Ravanona 2015; Siliconreview Team 2017), performing industrial strain engineering with heavy use of robotics, next generation sequencing, automation and software. Some of these companies like Ginkgo, are spin-off companies from universities seeking to capitalize on their proprietary technologies and in 2017 50 synthetic biology companies managed to raise 1.7 billion US dollars in funding (Calvin Schmidt 2018). Both academia and industry could benefit from continued and potential closer collaboration. Academia is well placed to investigate the basic biochemistry of the systems it engineers, furthering understanding of the relationship between circuit and host and do the groundwork that enables basic modular functional parts whilst industry works to apply the principles to relevant real-world applications. It would be pertinent for industry here to establish a forum for discussion of specific problems that need to be tackled for relevant market needs that academia could potentially cooperate on. Closer partnership will require adoption of model organisms that are more relevant for biotechnology and close collaboration with fields such as chemical engineering that work with relevant techniques in order to bridge the gap between proof of concept and industrially sized production (Moser et al. 2012).
